# Use of Bayesian approaches in oncology clinical trials: A cross-sectional analysis

**DOI:** 10.3389/fphar.2025.1548997

**Published:** 2025-03-25

**Authors:** Borja G. Lopez-Rey, Gerard Carot-Sans, Dan Ouchi, Ferran Torres, Caridad Pontes

**Affiliations:** ^1^ Spanish Agency of Medicines and Medical Devices (AEMPS), Madrid, Spain; ^2^ Biostatistics Unit, Medical School, Department of Paediatrics, Obstetrics and Gynaecology and Preventive Medicine and Public Health, Universitat Autònoma de Barcelona, Barcelona, Spain; ^3^ Catalan Health Service, Barcelona, Spain; ^4^ Digitalization for the Sustainability of the Healthcare System (DS3), Barcelona, Spain; ^5^ Departament de Farmacologia, de Toxicología i de Terapèutica, Universitat Autònoma de Barcelona, Barcelona, Spain; ^6^ Servei de Farmacologia Clínica, Hospital de la Santa Creu i de Sant Pau, Barcelona, Spain

**Keywords:** Bayesian approach, oncology clinical trials, cross-sectional study, small sample size, single-arm design, external data, decision-making

## Abstract

**Purpose:**

Bayesian approaches may improve the efficiency of trials and accelerate decision-making, but reluctance to depart from traditional frequentist statistics may limit their use. Because oncology trials generally involve severe conditions with no or limited therapeutic options, they are well-suited to applying Bayesian methodologies and are perceived as using these methods often in early phases.

**Objectives:**

In this study, we aim to describe the use of Bayesian methods and designs in oncology clinical trials in the last 20 years.

**Method:**

A cross-sectional observational study was conducted to identify oncology clinical trials using Bayesian approaches registered in clinicaltrials.gov between 2004 and 2024. Trials were searched in clinicaltrials.gov, PubMed, and through manual search of cross-references.

**Results:**

Bayesian trials were retrieved, and their main characteristics were extracted using R and verified manually. Between 2004 and 2024, 384,298 trials were registered in clinicaltrials.gov; we identified 84,850 oncology clinical trials (22%), of which 640 (0.75%) used Bayesian approaches. The adoption of Bayesian trials increased significantly after 2011, but while half of all Bayesian studies started in the last 5 years, this paralleled the overall increase in oncology research rather than an increase in the proportion of Bayesian trials. The majority of Bayesian trials were phase 1 and phase 2 studies, and two-thirds of Bayesian trials with efficacy objectives had single-arm designs, often utilizing binary endpoints, such as overall response, as the primary measure.

**Conclusion:**

The uptake of Bayesian methods in oncology clinical trials has flattened and is still scarce, and is mostly applied to the analysis of treatment efficacy in single-arm trials with binary endpoints. There is room for further uptake and use of their potential advantages in settings with small populations and severe conditions with unmet needs.

## Introduction

The traditional drug development pathway is often slow, usually taking more than 10 years to reach clinical practice, with the longest period being clinical research (The Institute of Cancer Research, 2024). This process is not only time-consuming but also expensive and risky for sponsors (DiMasi et al., 2016; Takebe et al., 2018; Dowden and Munro, 2019; Sun et al., 2022). Furthermore, challenges such as patient recruitment and ethical concerns can hamper clinical development in certain contexts. One way to accelerate access to new treatments is to use innovative and efficient trial designs that go beyond the traditional randomized parallel design (Halperin and Ware, 1990; Bhatt and Mehta, 2015; Bauer et al., 2016; Prowell, et al., 2016; Renfro and Mandrekar, 2018).

In addition to the use of innovative designs, Bayesian statistics is a viable alternative for drawing relevant conclusions in confirmatory trials. Bayesian approaches offer several advantages over traditional frequentist methods, such as providing more information to decision-makers and incorporating prior information that may reduce the need for larger trial sizes (Food and Drug Administration, 2010; Gupta, 2012; Jack Lee and Chu, 2012; Schmidli et al., 2014; Hummel et al., 2015; Spiegelhalter et al., 2015; Ryan et al., 2019).

To date, Bayesian methods have been reported to be mainly applied with the aim of reducing trial sample size in cancer (Sridhara et al., 2015; Blagden et al., 2020), rare diseases (Gupta et al., 2011; Bogaerts et al., 2015; Abrahamyan et al., 2016; Goring et al., 2019; Partington et al., 2022; Mackay and Springford, 2023), and pediatric trials (Gamalo-Siebers et al., 2017; Huff et al., 2017; European Medicines Agency, 2018; Travis, et al., 2023), all areas where unmet medical needs are often related to severe conditions, lack of alternative effective treatments, and vulnerable populations. These methods are thus particularly advantageous in oncology, where clinical trials, on average, take twice as long to complete compared to non-oncology trials (Wong et al., 2019), with lower success rates (Thomas et al., 2016; Dowden and Munro, 2019; Wong et al., 2019) and greater complexity due to the heterogeneity of cancer (Wang et al., 2017). Frequentist approaches confirm or reject a null hypothesis based on the probability of obtaining a result equal to or more extreme than the one observed, assuming the null hypothesis is true. While frequentist methods are widely accepted as robust and objective, they can also present significant limitations, such as inflexible study designs and clinically less intuitive interpretations due to their binary conclusions regarding the likelihood of a given result. In contrast, Bayesian approaches utilize prior distributions to estimate the probability of causality by integrating prior knowledge with new data, thereby mimicking the natural flow of clinical reasoning (Labos and Thanassoulis, 2020). Moreover, Bayesian methods allow for adaptive trial designs, enabling modifications to study parameters as data accumulate. This flexibility can enhance trial efficiency and minimize unnecessary patient exposure to potentially suboptimal clinical interventions (Thall and Wathen, 2007). Despite these advantages, which may be particularly relevant in exploratory settings or when limited sample size threatens statistical power, preliminary data suggest that Bayesian trials are not widely used (Pontes et al., 2018).

The objective of this cross-sectional observational study was to quantify the use of Bayesian methods and designs in oncology clinical trials and to describe the characteristics of Bayesian trials to identify further opportunities to improve their use.

## Materials and methods

### Overall design and information sources

This cross-sectional study included all the clinical trials registered in clinicaltrials.gov between 1 January 2004 and 1 October 2024 that investigated an oncological condition. From these, we selected those that used Bayesian design methodologies for characterization. The identification of Bayesian oncology trials was complemented with peer-reviewed articles reporting trial results. We searched PubMed for articles meeting these criteria and screened their reference lists for additional potentially eligible clinical trials. From the selected publications, NCT numbers were extracted and cross-checked for duplicates against the primary search on clinicaltrials.gov.

The search strategies are detailed in Supplementary Data Sheet 1. Briefly, we searched for interventional studies with any of the following clinical terms: cancer, oncology, tumor, tumour, neoplasm, immunotherapy, carcinoma, sarcoma, lymphoma, leukemia, myelodysplastic syndrome, blastoma, melanoma, neoplasia, myeloma, glioma, and among these, registries with at least one of the following descriptors: Bayes, prior distribution, posterior distribution, Bayesian, credible interval, mixture prior, power prior, BOIN (Bayesian optimal interval), Bayesian hierarchical model, or BLRM (Bayesian logistic regression model). Searches were conducted through 1 October 2024. Data analysis was completed in November 2024.

### Eligibility criteria

Inclusion criteria were clinical trials with the first available record in the clinicaltrials.gov database between 1st January 2004 and 1st October 2024, and articles on human interventional clinical trials in oncology using Bayesian methodologies published between 1st January 2004 and 1st October 2024 and having an associated NCT identifier. Exclusion criteria were articles written in a language other than English, and reviews including information unrelated to human interventional clinical trials, such as veterinary studies, retrospective studies, case studies, or meta-analyses. Duplicates were identified through the NCT identifier and removed as required. Only publicly available data on clinical trials were used.

The study followed the Strengthening the Reporting of Observational Studies in Epidemiology guidelines (von Elm et al., 2007) and the checklist for cross-sectional studies.

### Data extraction

Trial descriptors, relevant protocol, and result-related information were extracted from the clinicaltrials.gov database using the ctrdata package (Herold, 2024) in the R environment [version 4.2.3, (R Core Team, 2018)]. Trial descriptors included the following: clinical phase, year of start, year of publication of the first record in clinicaltrials.gov, sample size, status, number of arms, number of patients, primary and secondary objectives of the trial, identification of Bayesian analysis for primary or secondary endpoints, type of endpoints and variables for primary and secondary objectives, and therapeutic indication. For several categories, such as trial objectives and endpoints, the definitions were not mutually exclusive. The information from each clinical trial was manually reviewed to confirm accuracy and complemented with information from full publications and Supplementary Materials as needed, for example, when data were missing in the predefined clinicatrials.gov fields. Details of the automatically extracted fields and manually included fields are listed in the Supplementary Data Sheet 1.

### Statistical analysis

Descriptive statistics were used to summarize trial characteristics. Categorical variables were reported as frequencies and percentages, and continuous variables were categorized into ranges (i.e., number of arms and trial sample size) and described accordingly. No formal hypothesis testing was conducted.

## Results

### Number of Bayesian clinical trials

Our search of clinicaltrials.gov yielded 84,850 oncology trials conducted between 1 January 2004 and 1 October 2024. Of these, 538 were identified as using Bayesian methodologies using search terms in the clinicaltrials.gov database. The number of trials was enriched with trials identified in PubMed articles and their corresponding reference lists, resulting in a total of 640 (640/84,850; 0.75%) oncology trials that used Bayesian methodologies and had an NCT identifier (eFigure1, Supplementary Data Sheet 1).

**FIGURE 1:**
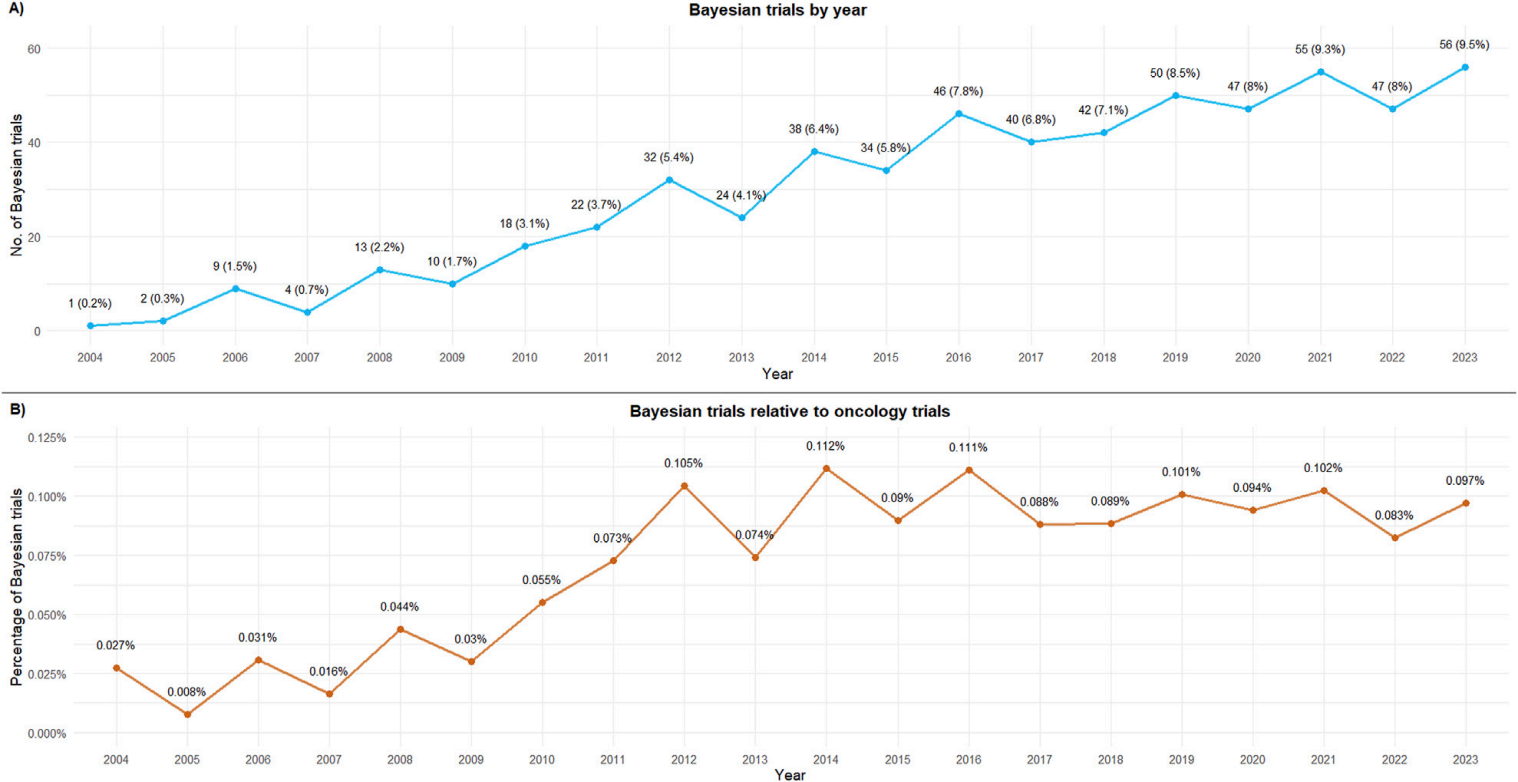
Bayesian trials by first published year. **(A)** Bayesian trials by year; **(B)** Bayesian trials relative to oncology trials. 2024 has been excluded from the figure as data were available only until October 2024.

**FIGURE 2:**
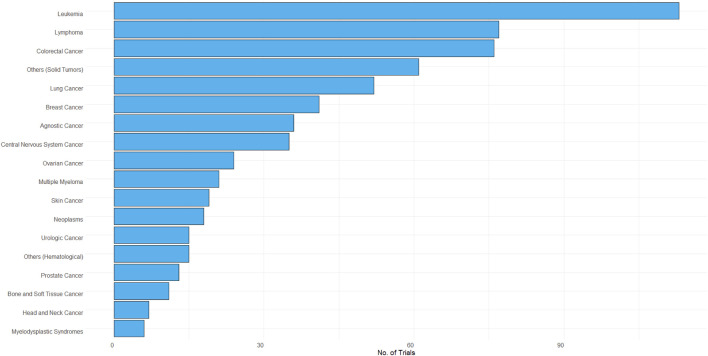
Bayesian trials by cancer type.

The proportion of oncology studies that implemented Bayesian methods increased in 2011 and has remained relatively constant since then (Figure 1). The majority of Bayesian trials included hematological indications (Figure 2).

### Characteristics of Bayesian oncology clinical trials

Bayesian methods/designs were most commonly used in phase 1 (n = 263; 41.1%) and phase 2 trials (n = 215; 33.6%). A total of 23 trials used Bayesian designs in confirmatory phases: [9 (1.4%) in phase 2/3 trials and 14 (2.2%) in phase 3]. The majority of oncology Bayesian trials were single-arm designs (n = 388; 60.6%), of which 43.8% were phase 1 and 35.1% were phase 2. The most prevalent complex designs among oncology Bayesian trials were adaptive phase 1/2 trials (n = 35) and master protocol designs. Among the latter, there were seven umbrella trials, nine platform designs, and eight single-arm trials included as part of a Bayesian platform of basket designs. The most common enrollment range was between 11 and 30 patients per study. A single hospital center sponsored 212 (33.1%) of all oncology Bayesian trials, mostly phase 2 (61.3%); industry-sponsored another third [n = 200; 31.2%, primarily phase 1 trials (55.5%)]. Almost half of the trials were reported as still recruiting (n = 201; 31.4%) or ongoing (n = 105; 16.3%), while the remainder (n = 271; 42.3%) were reported as already completed (Table 1).

**TABLE 1:** Characteristics Bayesian trials in oncology, overall and by phase.

Bayesian clinical trial	Phase 1	Phase 1/2	Phase 2	Phase 2/3	Phase 3	Total
Number of studies, No. (%)	263 (41.1)	139 (21.7)	215 (33.6)	9 (1.4)	14 (2.2)	640
Sponsor types, No. (%)						
Industry	111 (55.5)	58 (29)	26 (13)	1 (0.5)	4 (2)	200
Academy	43 (44.3)	17 (17.5)	26 (26.8)	6 (6.2)	5 (5.2)	97
Hospital^a^	93 (29.4)	61 (19.3)	157 (49.7)	2 (0.6)	3 (0.9)	316
NIH^b^	16 (59.3)	3 (11.1)	6 (22.2)	0 (0)	2 (7.4)	27
Status, No. (%)^c^						
Pre-recruitment	25 (53.2)	12 (25.5)	10 (21.3)	0 (0)	0 (0)	47
Recruiting	81 (40.3)	57 (28.4)	53 (26.4)	7 (3.5)	3 (1.5)	201
Ongoing	40 (38.1)	13 (12.4)	50 (47.6)	0 (0)	2 (1.9)	105
Completed	109 (40.2)	55 (20.3)	97 (35.8)	2 (0.7)	8 (3)	271
Unknown status	8 (50)	2 (12.5)	5 (31.2)	0 (0)	1 (6.2)	16
Number of arms, No. (%)						
1	170 (43.8)	70 (18)	136 (35.1)	7 (1.8)	5 (1.3)	388
2	43 (35.2)	27 (22.1)	45 (36.9)	1 (0.8)	6 (4.9)	122
3	23 (39.7)	19 (32.8)	15 (25.9)	0 (0)	1 (1.7)	58
4	9 (31)	11 (37.9)	9 (31)	0 (0)	0 (0)	29
≥5	18 (41.9)	12 (27.9)	10 (23.3)	1 (2.3)	2 (4.7)	43
Number of patients, No. (%)						
0–10	28 (51.9)	9 (16.7)	17 (31.5)	0 (0)	0 (0)	54
11–20	51 (59.3)	12 (14)	21 (24.4)	1 (1.2)	1 (1.2)	86
21–30	47 (54.7)	13 (15.1)	20 (23.3)	6 (7)	0 (0)	86
31–40	34 (46.6)	9 (12.3)	30 (41.1)	0 (0)	0 (0)	73
41–50	24 (39.3)	16 (26.2)	20 (32.8)	0 (0)	1 (1.6)	61
51–70	26 (34.7)	19 (25.3)	28 (37.3)	0 (0)	2 (2.7)	75
71–100	24 (34.3)	19 (27.1)	27 (38.6)	0 (0)	0 (0)	70
101–150	10 (17.9)	20 (35.7)	25 (44.6)	0 (0)	1 (1.8)	56
151–200	7 (23.3)	10 (33.3)	12 (40)	0 (0)	1 (3.3)	30
201–300	8 (30.8)	10 (38.5)	5 (19.2)	1 (3.8)	2 (7.7)	26
>300	4 (17.4)	2 (8.7)	10 (43.5)	1 (4.3)	6 (26.1)	23
Trial objectives, No. (%)^d^						
Efficacy	194 (35.8)	124 (22.9)	208 (38.4)	8 (1.5)	8 (1.5)	542
Primary objective	32 (9.9)	88 (27.3)	188 (58.4)	8 (2.5)	6 (1.9)	322
Secondary objective	162 (73.6)	36 (16.4)	20 (9.1)	0 (0)	2 (0.9)	220
Safety/toxicity	201 (49.8)	91 (22.5)	103 (25.5)	6 (1.5)	3 (0.7)	404
Primary objective	152 (62.8)	56 (23.1)	33 (13.6)	0 (0)	1 (0.4)	242
Secondary objective	49 (30.2)	35 (21.6)	70 (43.2)	6 (3.7)	2 (1.2)	162
Dose-finding	229 (62.6)	115 (31.4)	19 (5.2)	1 (0.3)	2 (0.5)	366
Primary objective	223 (62.8)	111 (31.3)	18 (5.1)	1 (0.3)	2 (0.6)	355
Secondary objective	6 (54.5)	4 (36.4)	1 (9.1)	0 (0)	0 (0)	11
Others^e^	2 (10.5)	0 (0)	8 (42.1)	1 (5.3)	8 (42.1)	19
Primary objective	2 (10.5)	0 (0)	8 (42.1)	1 (5.3)	8 (42.1)	19
Implementation of Bayesian analysis, No. (%)						
Dose finding	211 (66.6)	91 (28.7)	13 (4.1)	1 (0.3)	1 (0.3)	317
Primary endpoint	205 (68.1)	87 (28.9)	8 (2.7)	1 (0.3)	0 (0)	301
Secondary endpoint	6 (37.5)	4 (25)	5 (31.2)	0 (0)	1 (6.2)	16
Efficacy	20 (10.1)	35 (17.7)	132 (66.7)	6 (3)	5 (2.5)	198
Primary endpoint	9 (5.6)	26 (16.2)	117 (73.1)	6 (3.8)	2 (1.2)	160
Secondary endpoint	11 (28.9)	9 (23.7)	15 (39.5)	0 (0)	3 (7.9)	38
Monitoring	10 (17.5)	5 (8.8)	42 (73.7)	0 (0)	0 (0)	57
Safety/toxicity	20 (45.5)	5 (11.4)	19 (43.2)	0 (0)	0 (0)	44
Primary endpoint	20 (62.5)	4 (12.5)	8 (25)	0 (0)	0 (0)	32
Secondary endpoint	0 (0)	1 (8.3)	11 (91.7)	0 (0)	0 (0)	12
Others^e^	2 (8.3)	3 (12.5)	9 (37.5)	2 (8.3)	8 (33.3)	24
Primary endpoint	1 (5.3)	3 (15.8)	7 (36.8)	2 (10.5)	6 (31.5)	19
Secondary endpoint	1 (20)	0 (0)	2 (40)	0 (0)	2 (40)	5

^a^ Of the hospital-sponsored trials, 67.1% (212) were sponsored by a single sponsor (M.D., Anderson Cancer Center), and included 39 phase 1 trials, 40 phase 1/2 trials, 130 phase 2 trials, and 3 phase 3 trials.

^b^ NIH: National Institutes of Health.

^c^ Status as of 1 October 2024.

^d^ Trials may involve multiple objectives; hence, totals may exceed the number of trials.

^e^ Others include objectives such as diagnosis, screening, and prevention.

Efficacy was included as an objective in 542 Bayesian oncology trials [208/542 (38.4%) in phase 2 trials and 194/542 (35.8%) in phase 1 trials]; it was the primary objective in 90.3% (188/208) phase 2 trials and 16.4% (32/194) of phase 1 trials.

Safety/toxicity was included as an objective in 404 oncology Bayesian trials; it was the primary objective in 75.6% (152/201) of all Bayesian oncology phase 1 trials. Dose-finding objectives were present in 366 Bayesian oncology trials, with 62.6% (229/366) being phase 1 studies and 31.4% (115/366) phase 1/2 studies. Among the trials that implemented Bayesian analysis, this was primarily used for dose-finding (n = 317; 49.5%), and mostly when used as a primary endpoint in phase 1 (Table 1).

### Bayesian oncology clinical trials assessing efficacy

Efficacy was analyzed through Bayesian approaches in 198 trials, mostly in phase 2 trials (132/198; 66.7%), and, among these, mostly as the main analysis of the primary endpoint (117/132; 88.6%). In confirmatory phases, Bayesian analysis of efficacy was applied to 5.5% of the trials (Table 1). Of the 198 trials that used Bayesian analysis for efficacy assessment, 126 trials (63.6%) were single-arm trials, and 41 (20.7%) had two-arm designs. Binary variables were used more frequently (118 trials; 59.6%) than time-to-event variables (63 trials; 31.8%) and continuous variables (17 trials; 8.6%). Binary assessments of tumor response were the most frequent type of primary endpoint (61.6%), followed by progression-free survival or similar time-to-event-based endpoints (25.8%). Overall survival was assessed as a secondary endpoint in 60.1% of the trials (Table 2).

**TABLE 2:** Characteristics of oncology Bayesian trials assessing efficacy.

Bayesian clinical trial	No. (%)
Number of studies	198 (100)
No of arms	
1	126 (63.6)
2	41 (20.7)
3	14 (7.1)
4	6 (3)
≥5	11 (5.6)
Endpoint types^a^	
Response as the primary endpoint	122 (61.6)
Progression-/recurrence-/relapse-/disease-/event-free survival as primary endpoint	51 (25.8)
Overall survival as the primary endpoint	18 (9.1)
Overall survival as the secondary endpoint	119 (60.1)
Variable types for Bayesian analysis	
Binary	118 (59.6)
Time-to-event	63 (31.8)
Continuous	17 (8.6)
Bayesian complex designs	
Adaptive phase 1/2 design	35 (17.7)
Adaptive phase 2/3 design	6 (3)
Bayesian umbrella design	7 (3.5)
Bayesian MAMs^b^ design	1 (0.5)
Bayesian platform design	9 (4.5)
Bayesian platform of basket designs	8 (4)

^a^ Trials may involve multiple endpoints; hence, totals can exceed the number of trials.

^b^ MAMs: multi-arm multi-stage trials.

The sample size was generally higher in trials with primary events based on time-to-event endpoints (eFigure2, Supplementary Data Sheet 1).

## Discussion

This cross-sectional analysis of oncology clinical trials using Bayesian approaches reveals an upward trend in the adoption of these methods in absolute terms, with 50% of all trials registered on clinicaltrials.gov using these methodologies being conducted in the last 5 years. However, this increase likely reflects the overall rise in oncology trials rather than a specific preference for Bayesian methods, as shown by the steady profile of proportions over this period.

Our data indicate an uptick in Bayesian trial registrations from 2010 to 2012, coinciding with the release of the first regulatory guidance on Bayesian designs (although applied to medical devices). The draft guidance available in 2007 and its final version in 2010 (Food and Drug Administration, 2010) may have contributed to increased awareness and adoption of these methods. However, from 2012 onward, the trend appears to be relatively flat and constant, with some fluctuations between years. While some previous reports have suggested a rising adoption of these methods (Golchi, 2022; Kidwell et al., 2022; Best et al., 2024), our study does not confirm this when considering the relative preference for Bayesian methods in relation to the total number of oncology trials.

Traditionally employed in exploratory phases for safety, toxicity monitoring, and dose-finding (Ashby, 2006; Gaydos et al., 2006; Biswas et al., 2009; Chevret, 2012; Jack Lee and Chu, 2012; Bhattacharjee, 2014; Tidwell et al., 2019), our data indicate that Bayesian methods are now increasingly used to address efficacy objectives. Bayesian analyses are primarily used for dose-finding, particularly in phase 1 studies and as part of the efficacy analysis for the primary endpoint in phase 2 studies. This aligns with previous reports suggesting that these methods are perceived as a valuable solution in complex scenarios, such as those lacking effective alternatives and with challenging patient recruitment, when classical randomized trials are impractical, or when surrogate endpoints may be unreliable (Prasad and Oseran, 2015). Bayesian methods are increasingly used in complex settings like adaptive designs (Morgan et al., 2014; FDA, 2019; Ben-Eltriki et al., 2024), pediatric studies (Gamalo-Siebers et al., 2017; Huff et al., 2017; European Medicines Agency, 2018; Travis et al., 2023), and rare diseases (Gupta et al., 2011; Bogaerts et al., 2015; Goring et al., 2019; Partington et al., 2022; Mackay and Springford, 2023). Additionally, their frequent use in innovative trial frameworks such as master protocols (Berry, 2015; Renfro and Sargent, 2017; Simon, 2017; Woodcock and LaVange, 2017; Hirakawa et al., 2018; Meyer et al., 2020; Food and Drug Administration FDA, 2022; Quintana et al., 2023) leverages their flexibility and effectiveness in uncertain research scenarios.

Overall, oncology is deemed to be the area with greater adoption of the Bayesian framework (Sridhara et al., 2015; Blagden et al., 2020). Although determining the proportion of all Bayesian studies that are implemented in oncology was not an objective of our study, when our filters for Bayesian descriptors were applied to the clinicaltrials.gov database for interventional studies before selecting oncological conditions, 879 records were retrieved out of the 384,298 records (0.23%) for the study period, resulting in 61.2% (538/879) of all Bayesian designs identified at this step being in the oncology setting. The higher acceptance and adoption of Bayesian designs in oncology may be due to several factors, led by the precedent of Bayesian methods traditionally applied to first-in-human studies with chemotherapeutics. Acceptability may also be related to a broader awareness of the benefits of using such an approach in adaptive designs, allowing decision-making informed by current data, incorporating external information to the previous distribution to inform treatment arms, bolster control data arms, or include external control arms. These may lead to potential gains in efficiency, either through adaptive designs or the use of external data, and thus to expedited drug development, shorter trial durations, more comprehensive information for decision-makers, smaller trial sizes, and overall lower costs (FDA, 2010; Gupta, 2012; Jack Lee and Chu, 2012; Schmidli et al., 2014; Hummel et al., 2015; Spiegelhalter et al., 2015; Ryan et al., 2019).

Advancements in computational challenges (Chevret, 2012; Bhattacharjee, 2014) and increased familiarity with Bayesian approaches among stakeholders are expected to gradually reduce reluctance from regulatory bodies and sponsors (European Medicines Agency, 2007; European Medicines Agency, 2024; Food and Drug Administration, 2017, Food and Drug Administration, 2019, Food and Drug Administration, 2020). However, reliance on priors and their potential impact on trial outcomes remain significant barriers, particularly in confirmatory settings where unbiased and reproducible results are critical. This may explain why, in our study, Bayesian trials generally involve small sample sizes associated with phase 1 and 2 stages, typically consisting of fewer than 150 patients, with only a few trials featuring larger sample sizes.

While our analysis highlights the significant role of hospitals and academia in implementing Bayesian designs in exploratory settings, the industry is also widely sponsoring Bayesian trials, mostly in phase 1 and phase 1/2 studies, as already described previously (Califf et al., 2012; Fors and González, 2020). This uptake by industry sponsors in early development with exploratory objectives may reflect the usefulness of these methods for decision-making, whereas the relatively low uptake in confirmatory development may indicate a reluctance to assume the regulatory risk associated with non-frequentist designs.

Of note, we observed that Bayesian methods were primarily applied to binary endpoints when sample sizes were small, regardless of the number of trial arms, whereas those with more than 100–150 patients generally relied on time-to-event variables. In addition, small sample sizes were paralleled by a high proportion of single-arm trials, a design that may further hamper the methodological robustness of clinical studies and the generation of solid evidence (Pontes et al., 2018). The use of Bayesian approaches in these trials reflects that one of the main advantages of Bayesian designs is that they can add value, particularly when there is limited availability of potential candidates for trial recruitment, by incorporating external information into the prior distribution when assessing efficacy, thus allowing for a more robust design than designs based solely on a single-arm strategy (Viele et al., 2014; FDA, 2020; Mackay and Springford, 2023).

Furthermore, the frequent use of binary endpoints in Bayesian approaches highlights that these variables allow gathering evidence more quickly than time-to-event endpoints, which typically require longer follow-ups and larger sample sizes.

Despite the aforementioned advantages, we observed that Bayesian approaches accounted only for 0.75% of all oncology clinical trials, which appears to be a missed opportunity. The reasons for this may be related to regulatory reluctance to depart from classical standards so that even in cases where such designs are feasible, regulatory bodies may be prone to request a frequentist approach rather than a Bayesian one. Given the efficiency advantages that a Bayesian approach can offer (Food and Drug Administration, 2010; Gupta, 2012; Jack Lee and Chu, 2012; Schmidli et al., 2014; Hummel et al., 2015; Spiegelhalter et al., 2015; Ryan et al., 2019), it is reasonable that these methods could be more widely accepted and promoted, especially in clinical settings where obtaining evidence is challenging (Chevret, 2012).

For instance, the Bayesian framework facilitates the integration of information from earlier trial phases or previous studies into the current analysis using prior distributions. This capability is frequently highlighted as a key practical advantage, as it enables ongoing optimization of the trial design within predefined rules. The inherent flexibility of Bayesian methods makes them particularly valuable in contexts requiring efficiency improvements, such as potentially reducing sample size requirements, while maintaining the reliability of clinical trial results, provided that appropriate statistical operating characteristics are established before trial initiation (Wang, 2007; Veenman et al., 2024). These advantages prove to be useful in the following situations: a) when sample sizes are small and patient recruitment is challenging, b) when assigning patients to negative control arms may be logistically or ethically complex (e.g., in severe conditions or when studying highly vulnerable populations), c) when there is a need for swift adjustments in response to an evolving disease (Garczarek et al., 2023) and dynamic environment (Polack et al., 2020; Ruberg et al., 2023), and d) in dose-finding settings (Garrett-Mayer, 2006).

Additionally, Bayesian approaches offer direct probabilistic interpretations of parameters (e.g., “there is a 95% probability that the parameter lies within this range”), which are often more intuitive for decision-makers. In contrast, frequentist methods, such as p-values or confidence intervals, do not provide such straightforward probabilistic interpretations. Although confidence intervals are widely used, they are frequently misunderstood as probabilistic statements about the parameter. In fact, they indicate the range that would encompass the true parameter in 95% of repeated samples under identical conditions. This distinction makes Bayesian methods particularly valuable in clinical and regulatory contexts, where clear and actionable insights are critical (Greenland et al., 2016; Ryan et al., 2019). By providing increased flexibility and taking advantage of incremental learning (Schönbrodt et al., 2017), Bayesian approaches follow the same logic of continuous and evolving human learning, which may prove especially useful in early clinical development and exploratory research (Labos and Thanassoulis, 2020).

Currently, the European Medicines Agency is sponsoring an initiative to increase the applicability and acceptability of Bayesian approaches in clinical trials (European Medicines Agency, 2024). Bayesian approaches are particularly relevant to oncology research due to their ability to address challenges in patient recruitment, seamlessly integrate data from multiple sources, and adapt dynamically through their inherent learning capabilities. Consequently, there is significant potential to expand their application in the oncology field (Food and Drug Administration, 2010; Gupta, 2012; Jack Lee and Chu, 2012; Schmidli et al., 2014; Hummel et al., 2015; Spiegelhalter et al., 2015; Ryan et al., 2019; European Medicines Agency, 2024). With progress in targeted and advanced therapies (Sridhara et al., 2015; Nass et al., 2018), their increasing potential in the design of oncology clinical trials should be relevant in the evolving landscape of cancer treatment research.

### Limitations

This analysis has limitations. First, it depends on the accurate self-identification of Bayesian designs in trial registries, which may overlook studies using Bayesian methods that are described differently. In addition, trial features may not always be updated in databases following protocol amendments. However, the complementary literature search for NCT-identified oncology trials with Bayesian characteristics has likely compensated, at least partially, for this limitation. Furthermore, the classification of trials by phase, objective, and endpoint type relies on subjective interpretation, particularly when registry details are sparse. Finally, results may be underestimated if relevant studies were not identified through our search parameters, or remained unpublished, thus escaping inclusion in the databases we reviewed.

## Conclusion

This cross-sectional study reveals that Bayesian methods are infrequently used in oncology trials, with the majority of implementations occurring in phase 1 and phase 2 studies. Notably, two-thirds of Bayesian trials with efficacy objectives are single-arm designs, often utilizing binary endpoints, such as overall response, as the primary measure.

Despite their potential to enhance the drug development process by providing a structured framework to improve trial efficiency, optimize decision-making, and incorporate external data, the adoption of Bayesian methods has remained limited in recent years. This indicates untapped potential for broader implementation, particularly in areas where traditional approaches have limitations.

Future efforts should address barriers to adoption, such as regulatory hesitancy and computational challenges while promoting education and stakeholder familiarity with Bayesian methodologies. By overcoming these hurdles, Bayesian approaches could play a more prominent role in transforming the design and execution of clinical trials.

## Data Availability

The raw data supporting the conclusions of this article will be made available by the authors, without undue reservation.
