# Supplementary material for: Use of Bayesian approaches in oncology clinical trials: A cross-sectional analysis
Source: Front Pharmacol. 2025 Mar 25;16:1548997. doi: 10.3389/fphar.2025.1548997 (PMC11975924; doi:10.3389/fphar.2025.1548997)
Supplement: Supplementary file 2 [file DataSheet1.docx]

**Supplementary materials**

Content

[Methods 2](#_Toc190094992)

[Eligibility Criteria 2](#_Toc190094993)

[Search Strategy 2](#_Toc190094994)

[1) Search strategy for clinicaltrials.gov: 2](#_Toc190094995)

[2) Search strategy for PubMed: 2](#_Toc190094996)

[Screening Strategy 3](#_Toc190094997)

[Data extraction 4](#_Toc190094998)

[Clinicaltrials.gov Fields 4](#_Toc190094999)

[Supplementary Figures and Tables 5](#_Toc190095000)

[eFigure1. PRISMA Diagram Detailing Study Selection 5](#_Toc190095254)

[eFigure2. Bayesian trials assessing efficacy in primary endpoints by arms, type of variables and enrollment. 6](#_Toc190095255)

[eTable 1. Characteristics of oncology Bayesian trials, overall, and by phase and cancer type. 7](#_Toc190095256)

[Table 2. Characteristics of oncology Bayesian trials, overall, and by phase and cancer type (2019-2024). 8](#_Toc190095257)

## Methods

### Eligibility Criteria

#### Eligibility criteria for clinicaltrials.gov:

**Condition/disease:** cancer OR oncology OR tumor OR tumour OR neoplasm OR immunotherapy OR carcinoma OR sarcoma OR lymphoma OR leukemia OR "myelodysplastic syndrome" OR blastoma OR melanoma OR neoplasia OR myeloma OR glioma.

**Other terms:** bayes OR bayesian OR "credible interval" OR "credible intervals" OR "prior distribution" OR "posterior distribution" OR BOIN OR BLRM OR "posterior probability" OR "prior information" OR MCMC OR "mixture prior" OR "power prior" OR "bayesian hierarchical model".

**Study type:** Interventional.

**First posted (The date on which the study record was first available on ClinicalTrials.gov after National Library of Medicine (NLM) quality control (QC) review has concluded):** 01/01/2004 - 01/10/2024.

#### Eligibility criteria for PubMed:

**[Title/Abstract]:** "bayes*" OR "prior distribution*" OR "posterior distribution*" OR bayesian OR "credible interval*" OR "mixture prior*" OR "power prior*" OR BOIN OR "bayesian hierarchical model*" OR BLRM) AND ("cancer*" OR oncology OR "tumor*" OR "neoplasm*" OR immunotherapy OR leukemia OR carcinoma OR sarcoma OR lymphoma OR "tumour*" OR blastoma OR melanoma OR neoplasia OR mieloma OR glioma).

AND

**[Filter]:** clinicaltrial OR randomizedcontrolledtrial) AND Clinical Trial, Randomized Controlled Trial, Humans.

**[From]:** 2004/1/1 - 2024/10/1.

## Search Strategy

### Search strategy for clinicaltrials.gov:

*“cancer OR oncology OR tumor OR tumour OR neoplasm OR immunotherapy OR carcinoma OR sarcoma OR lymphoma OR leukemia OR "myelodysplastic syndrome" OR blastoma OR melanoma OR neoplasia OR myeloma OR glioma | Other terms: bayes OR bayesian OR "credible interval" OR "credible intervals" OR "prior distribution" OR "posterior distribution" OR BOIN OR BLRM OR "posterior probability" OR "prior information" OR MCMC OR "mixture prior" OR "power prior" OR "bayesian hierarchical model" | Interventional studies | First posted from 01/01/2004 to 10/01/2024”*

Link: https://clinicaltrials.gov/search?cond=cancer%20OR%20oncology%20OR%20tumor%20OR%20tumour%20OR%20neoplasm%20OR%20immunotherapy%20OR%20carcinoma%20OR%20sarcoma%20OR%20lymphoma%20OR%20leukemia%20OR%20%22myelodysplastic%20syndrome%22%20OR%20blastoma%20OR%20melanoma%20OR%20neoplasia%20OR%20myeloma%20OR%20glioma&term=bayes%20OR%20bayesian%20OR%20%22credible%20interval%22%20OR%20%22credible%20intervals%22%20OR%20%22prior%20distribution%22%20OR%20%22posterior%20distribution%22%20OR%20BOIN%20OR%20BLRM%20%20OR%20%22posterior%20probability%22%20OR%20%22prior%20information%22%20OR%20MCMC%20OR%20%22mixture%20prior%22%20OR%20%22power%20prior%22%20OR%20%22bayesian%20hierarchical%20model%22%20&aggFilters=studyType:int&firstPost=2004-01-01_2024-10-01

### Search strategy for PubMed:

*“("bayes*"[Title/Abstract] OR "prior distribution*"[Title/Abstract] OR "posterior distribution*"[Title/Abstract] OR bayesian[Title/Abstract] OR "credible interval*"[Title/Abstract] OR "mixture prior*"[Title/Abstract] OR "power prior*"[Title/Abstract] OR BOIN[Title/Abstract] OR "bayesian hierarchical model*"[Title/Abstract] OR BLRM[Title/Abstract]) AND ("cancer*"[Title/Abstract] OR oncology[Title/Abstract] OR "tumor*"[Title/Abstract] OR "neoplasm*"[Title/Abstract] OR immunotherapy[Title/Abstract] OR leukemia[Title/Abstract] OR carcinoma[Title/Abstract] OR sarcoma[Title/Abstract] OR lymphoma[Title/Abstract] OR "tumour*"[Title/Abstract] OR blastoma[Title/Abstract] OR melanoma[Title/Abstract] OR neoplasia[Title/Abstract] OR myeloma[Title/Abstract] OR glioma[Title/Abstract]) AND (clinicaltrial[Filter] OR randomizedcontrolledtrial[Filter]) Filters: Clinical Trial, Randomized Controlled Trial, Humans, from 2004/1/1 - 2024/10/1 Sort by: Most Recent”*

Link: https://pubmed.ncbi.nlm.nih.gov/?term=%28%22bayes*%22%5BTitle%2FAbstract%5D+OR+%22prior+distribution*%22%5BTitle%2FAbstract%5D+OR+%22posterior+distribution*%22%5BTitle%2FAbstract%5D+OR+bayesian%5BTitle%2FAbstract%5D+OR+%22credible+interval*%22%5BTitle%2FAbstract%5D+OR+%22mixture+prior*%22%5BTitle%2FAbstract%5D+OR+%22power+prior*%22%5BTitle%2FAbstract%5D+OR+BOIN%5BTitle%2FAbstract%5D+OR+%22bayesian+hierarchical+model*%22%5BTitle%2FAbstract%5D+OR+BLRM%5BTitle%2FAbstract%5D%29+AND+%28%22cancer*%22%5BTitle%2FAbstract%5D+OR+oncology%5BTitle%2FAbstract%5D+OR+%22tumor*%22%5BTitle%2FAbstract%5D+OR+%22neoplasm*%22%5BTitle%2FAbstract%5D+OR+immunotherapy%5BTitle%2FAbstract%5D+OR+leukemia%5BTitle%2FAbstract%5D+OR+carcinoma%5BTitle%2FAbstract%5D+OR+sarcoma%5BTitle%2FAbstract%5D+OR+lymphoma%5BTitle%2FAbstract%5D+OR+%22tumour*%22%5BTitle%2FAbstract%5D+OR+blastoma%5BTitle%2FAbstract%5D+OR+melanoma%5BTitle%2FAbstract%5D+OR+neoplasia%5BTitle%2FAbstract%5D+OR+myeloma%5BTitle%2FAbstract%5D+OR+glioma%5BTitle%2FAbstract%5D%29+AND+%28clinicaltrial%5BFilter%5D+OR+randomizedcontrolledtrial%5BFilter%5D%29&filter=pubt.clinicaltrial&filter=pubt.randomizedcontrolledtrial&filter=dates.2004%2F1%2F1-2024%2F10%2F1&filter=hum_ani.humans&sort=date&size=200

## Screening Strategy

To identify Bayesian methods and designs in oncology trials that met our inclusion/exclusion criteria, we first searched the clinicaltrials.gov database using predefined terms related to Bayesian approaches and cancer. Since Bayesian methods are not always explicitly mentioned in clinicaltrials.gov records, we extended our search to PubMed to identify additional trials by locating their National Clinical Trial (NCT) numbers. This allowed us to include trials that used Bayesian approaches but were not explicitly indicated in clinicaltrials.gov. The same search terms were used in PubMed titles and abstracts as in the direct search of clinicaltrials.gov. Detailed search strategies for both databases are provided in the Supplement section.

Furthermore, we reviewed narrative and systematic reviews focused on topics such as oncology, CID trials, master protocols, and Bayesian designs. This helped us identify additional NCT numbers for trials not captured in the initial searches. The searches were conducted up to October 1, 2024.

After identifying all NCT numbers from different sources, relevant protocol- and results-related information for each clinical trial was automatically extracted from the clinicaltrials.gov database using the ctrdata package (Herold, 2024)^[[1]](#footnote-1)^ in the R environment (version 4.2.3, R Core Team (R Core Team, 2018))^[[2]](#footnote-2)^. Once the trials were retrieved, duplicates were checked and removed. Each trial was then assessed for eligibility, and missing or unclear data in key fields were filled in or corrected.

Additionally, because certain aspects relevant to our review lacked sufficient detail, supplementary information beyond the automated extraction was collected to ensure a comprehensive assessment of the trials. All information was double-checked to ensure the accuracy of the newly retrieved data.

## Data extraction

### Clinicaltrials.gov Fields

#### Fields extracted automatically:


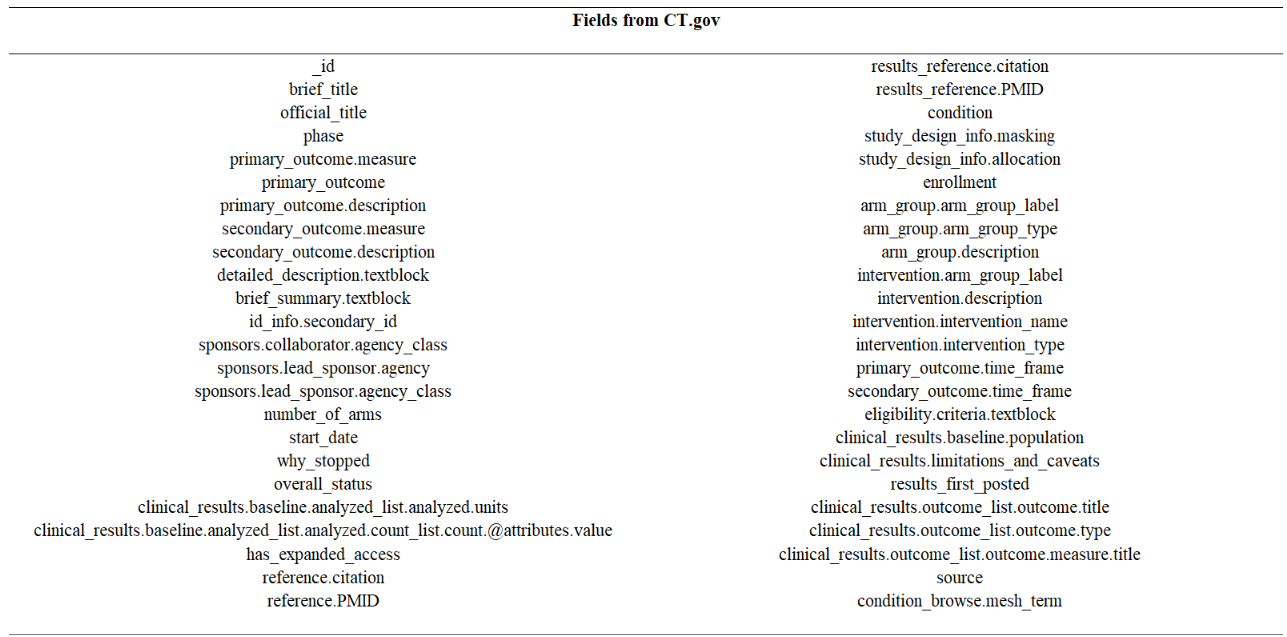


#### Fields created after manual review of each trial:


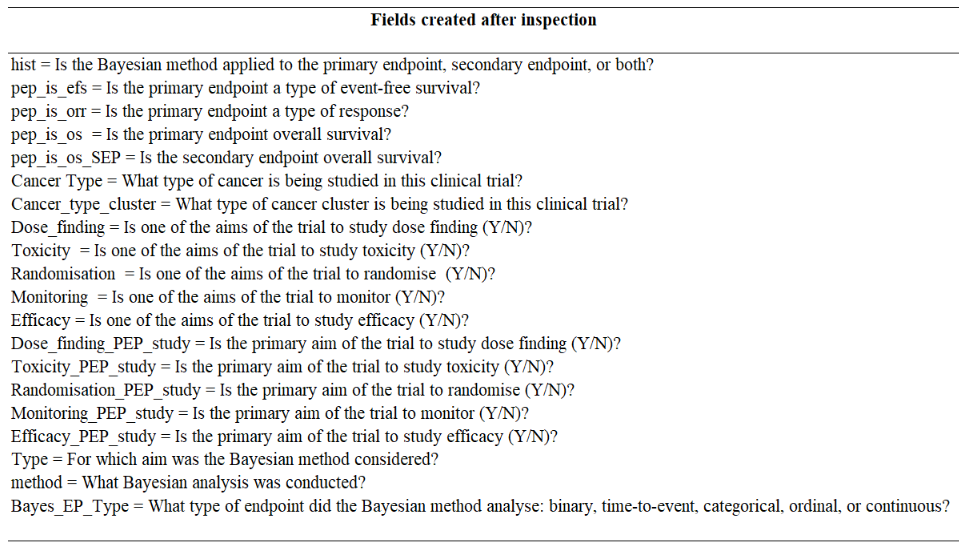


## Supplementary Figures and Tables

eFigure1. Flowchart Detailing Study Selection


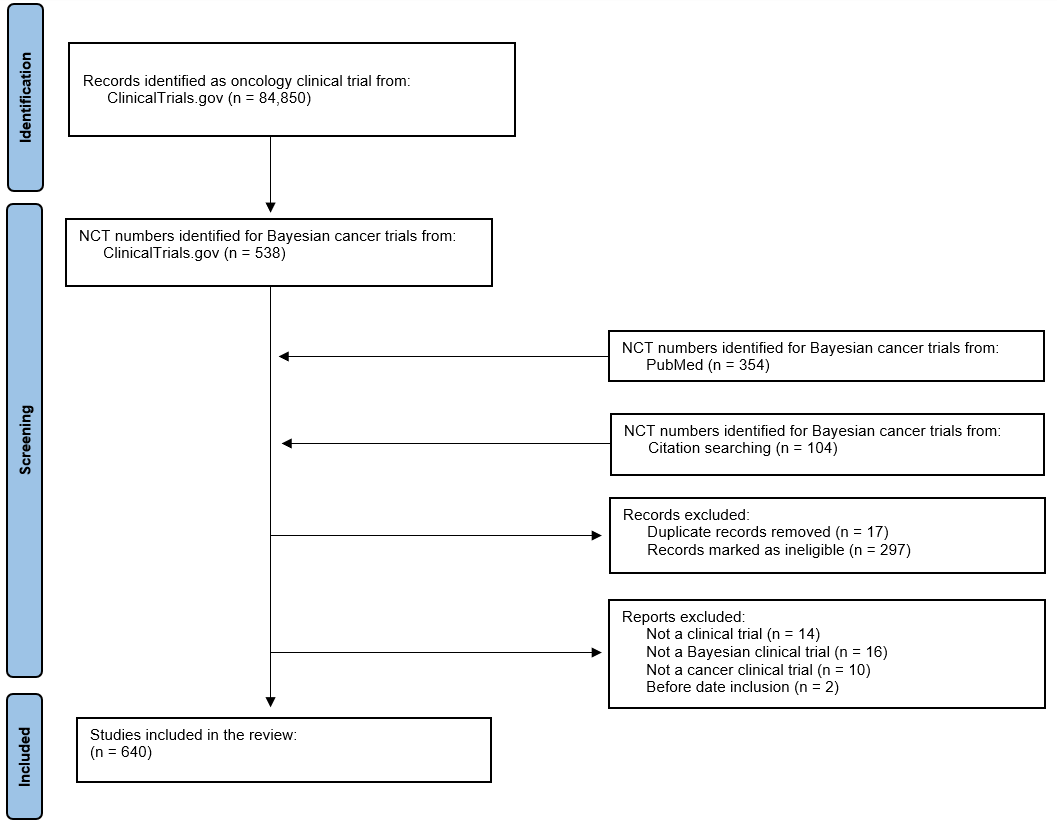


eFigure2. Bayesian trials assessing efficacy in primary endpoints by arms, type of variables and enrollment.

**
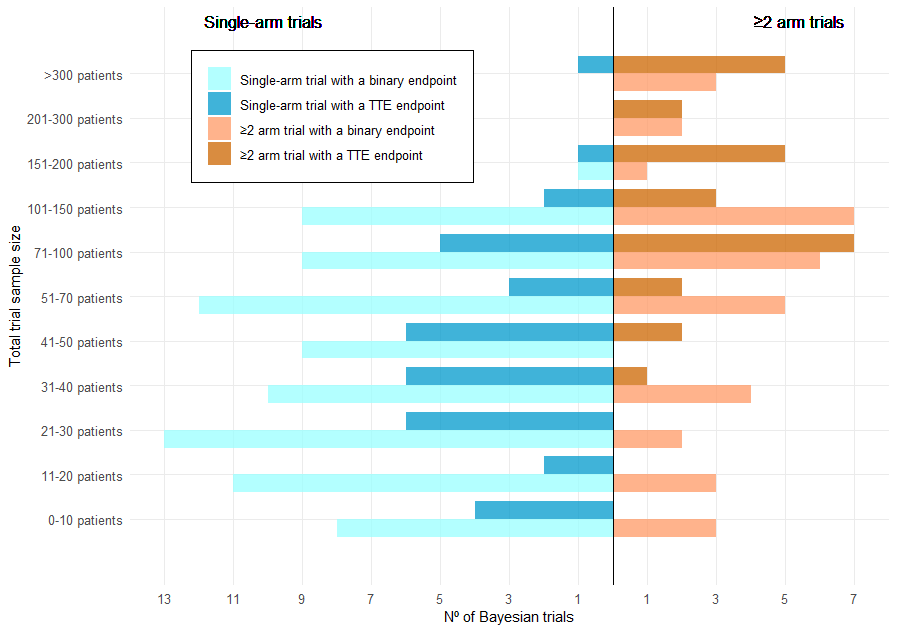
**

eTable 1. Characteristics of oncology Bayesian trials, overall, and by phase and cancer type.

| **Bayesian clinical trials** | **Phase 1** | **Phase 1/2** | **Phase 2** | **Phase 2/3** | **Phase 3** | **Total** |
| --- | --- | --- | --- | --- | --- | --- |
| **Number of studies** | 263 (41.1) | 139 (21.7) | 215 (33.6) | 9 (1.4) | 14 (2.2) | **640** |
| **Cancer types, No. (%)** |  |  |  |  |  |  |
| Agnostic Cancer | 16 (44.4) | 7 (19.4) | 8 (22.2) | 5 (13.9) | 0 (0) | **36** |
| Bone and Soft Tissue Cancer | 1 (9.1) | 2 (18.2) | 7 (63.6) | 0 (0) | 1 (9.1) | **11** |
| Breast Cancer | 17 (41.5) | 12 (29.3) | 10 (24.4) | 0 (0) | 2 (4.9) | **41** |
| Central Nervous System Cancer | 16 (45.7) | 10 (28.6) | 8 (22.9) | 1 (2.9) | 0 (0) | **35** |
| Colorectal Cancer | 33 (43.4) | 9 (11.8) | 29 (38.2) | 1 (1.3) | 4 (5.3) | **76** |
| Head and Neck Cancer | 1 (14.3) | 2 (28.6) | 4 (57.1) | 0 (0) | 0 (0) | **7** |
| Leukemia | 38 (33.6) | 31 (27.4) | 44 (38.9) | 0 (0) | 0 (0) | **113** |
| Lung Cancer | 17 (32.7) | 16 (30.8) | 17 (32.7) | 1 (1.9) | 1 (1.9) | **52** |
| Lymphoma | 30 (39) | 16 (20.8) | 30 (39) | 0 (0) | 1 (1.3) | **77** |
| Multiple Myeloma | 8 (38.1) | 6 (28.6) | 7 (33.3) | 0 (0) | 0 (0) | **21** |
| Myelodysplastic Syndromes | 0 (0) | 1 (16.7) | 4 (66.7) | 0 (0) | 1 (16.7) | **6** |
| Neoplasms | 8 (44.4) | 2 (11.1) | 6 (33.3) | 1 (5.6) | 1 (5.6) | **18** |
| Others (Hematological) | 5 (33.3) | 4 (26.7) | 6 (40) | 0 (0) | 0 (0) | **15** |
| Others (Solid Tumors) | 43 (70.5) | 12 (19.7) | 6 (9.8) | 0 (0) | 0 (0) | **61** |
| Ovarian Cancer | 14 (58.3) | 2 (8.3) | 6 (25) | 0 (0) | 2 (8.3) | **24** |
| Prostate Cancer | 5 (38.5) | 0 (0) | 7 (53.8) | 0 (0) | 1 (7.7) | **13** |
| Skin Cancer | 5 (26.3) | 5 (26.3) | 9 (47.4) | 0 (0) | 0 (0) | **19** |
| Urologic Cancer | 6 (40) | 2 (13.3) | 7 (46.7) | 0 (0) | 0 (0) | **15** |

eTable 2. Characteristics of oncology Bayesian trials, overall, and by phase and cancer type (2019-2024).

| **Bayesian clinical trials** | **Phase 1** | **Phase 1/2** | **Phase 2** | **Phase 2/3** | **Phase 3** | **Total** |
| --- | --- | --- | --- | --- | --- | --- |
| **Number of studies, No. (%)** | 157 (52.7) | 72 (24.2) | 60 (20.1) | 7 (2.3) | 2 (0.7) | **298** |
| **Cancer types, No. (%)*** |  |  |  |  |  |  |
| Agnostic Cancer | 6 (33.3) | 5 (27.8) | 2 (11.1) | 5 (27.8) | 0 (0) | **18** |
| Bone and Soft Tissue Cancer | 0 (0) | 2 (33.3) | 4 (66.7) | 0 (0) | 0 (0) | **6** |
| Breast Cancer | 8 (50) | 6 (37.5) | 2 (12.5) | 0 (0) | 0 (0) | **16** |
| Central Nervous System Cancer | 8 (61.5) | 2 (15.4) | 2 (15.4) | 1 (7.7) | 0 (0) | **13** |
| Colorectal Cancer | 22 (57.9) | 3 (7.9) | 10 (26.3) | 1 (2.6) | 2 (5.3) | **38** |
| Head and Neck Cancer | 0 (0) | 1 (100) | 0 (0) | 0 (0) | 0 (0) | **1** |
| Leukemia | 22 (39.3) | 20 (35.7) | 14 (25) | 0 (0) | 0 (0) | **56** |
| Lung Cancer | 14 (60.9) | 8 (34.8) | 1 (4.3) | 0 (0) | 0 (0) | **23** |
| Lymphoma | 17 (54.8) | 7 (22.6) | 7 (22.6) | 0 (0) | 0 (0) | **31** |
| Multiple Myeloma | 6 (54.5) | 2 (18.2) | 3 (27.3) | 0 (0) | 0 (0) | **11** |
| Neoplasms | 4 (80) | 0 (0) | 1 (20) | 0 (0) | 0 (0) | **5** |
| Others (Hematological) | 4 (44.4) | 2 (22.2) | 3 (33.3) | 0 (0) | 0 (0) | **9** |
| Others (Solid Tumors) | 26 (68.4) | 10 (26.3) | 2 (5.3) | 0 (0) | 0 (0) | **38** |
| Ovarian Cancer | 10 (90.9) | 1 (9.1) | 0 (0) | 0 (0) | 0 (0) | **11** |
| Prostate Cancer | 5 (62.5) | 0 (0) | 3 (37.5) | 0 (0) | 0 (0) | **8** |
| Skin Cancer | 1 (12.5) | 3 (37.5) | 4 (50) | 0 (0) | 0 (0) | **8** |
| Urologic Cancer | 4 (66.7) | 0 (0) | 2 (33.3) | 0 (0) | 0 (0) | **6** |
| Note*: Trials are displayed starting from the date of first posting in 2019. Unlike Table 1, where trials from 2024 are excluded, this table includes them. The number of trials is 255 for the period 2019-2023 and 43 for 2024. | | | | | | |

1. Herold, R. (2024) ‘ctrdata: Retrieve and Analyze Clinical Trials in Public Registers. R package version 1.17.1’. Available at: https://cran.r-project.org/package=ctrdata. [↑](#footnote-ref-1)
2. R Core Team (2018) ‘A language and environment for statistical computing. R Foundation for Statistical Computing, Vienna.’, Nature. Available at: https://www.r-project.org. [↑](#footnote-ref-2)
